# Automated saccharification assay for determination of digestibility in plant materials

**DOI:** 10.1186/1754-6834-3-23

**Published:** 2010-10-27

**Authors:** Leonardo D Gomez, Caragh Whitehead, Abdellah Barakate, Claire Halpin, Simon J McQueen-Mason

**Affiliations:** 1 CNAP, Department of Biology, University of York, Heslington, York YO10 5YW, UK; 2 Division of Plant Sciences, College of Life Sciences, University of Dundee at SCRI, Dundee, DD2 5DA, UK

## Abstract

**Background:**

Cell wall resistance represents the main barrier for the production of second generation biofuels. The deconstruction of lignocellulose can provide sugars for the production of fuels or other industrial products through fermentation. Understanding the biochemical basis of the recalcitrance of cell walls to digestion will allow development of more effective and cost efficient ways to produce sugars from biomass. One approach is to identify plant genes that play a role in biomass recalcitrance, using association genetics. Such an approach requires a robust and reliable high throughput (HT) assay for biomass digestibility, which can be used to screen the large numbers of samples involved in such studies.

**Results:**

We developed a HT saccharification assay based on a robotic platform that can carry out in a 96-well plate format the enzymatic digestion and quantification of the released sugars. The handling of the biomass powder for weighing and formatting into 96 wells is performed by a robotic station, where the plant material is ground, delivered to the desired well in the plates and weighed with a precision of 0.1 mg. Once the plates are loaded, an automated liquid handling platform delivers an optional mild pretreatment (< 100°C) followed by enzymatic hydrolysis of the biomass. Aliquots from the hydrolysis are then analyzed for the release of reducing sugar equivalents. The same platform can be used for the comparative evaluation of different enzymes and enzyme cocktails. The sensitivity and reliability of the platform was evaluated by measuring the saccharification of stems from lignin modified tobacco plants, and the results of automated and manual analyses compared.

**Conclusions:**

The automated assay systems are sensitive, robust and reliable. The system can reliably detect differences in the saccharification of plant tissues, and is able to process large number of samples with a minimum amount of human intervention. The automated system uncovered significant increases in the digestibility of certain lignin modified lines in a manner compatible with known effects of lignin modification on cell wall properties. We conclude that this automated assay platform is of sufficient sensitivity and reliability to undertake the screening of the large populations of plants necessary for mutant identification and genetic association studies.

## Introduction

Plant lignocellulosic biomass is widely considered to have the potential to reduce the world's reliance on petroleum for liquid transportation fuels and other chemicals, because it is cheap, abundant and contains energy rich polysaccharides that make up approximately 75% of its mass. In theory, these polysaccharides can be broken down to produce sugar substrates (saccharification) from which a range of useful products, such as biofuels, bioplastics, fine and bulk chemicals, food, and feed ingredients can be produced by fermentation [1]. The US Department of Energy has set a target of replacing 30% of petroleum consumption with biomass [2]. Even more value may be obtained by using integrated processing systems that allow multiple products to be produced from the same biomass feedstock: the biorefinery concept [3]. Perhaps the greatest barrier to realizing the potential of lignocellulose as an industrial feedstock lies in its indigestibility[4]. Cellulose, the major component of lignocellulose, is composed of polymers of pure glucose, but the crystalline nature of cellulose microfibrils makes this material resistant to both chemical and enzymatic degradation [5]. Cellulose microfibrils are embedded in matrix polysaccharides such as xylans and arabinoxylans, and the whole structure is interpenetrated and encased by the phenolic polymer lignin, another polymer that is difficult to digest [6,7]. In total, this composite material forms an insoluble macromolecular mass providing a challenge to degradation in either the biological or the industrial contexts. Improving the ease and yield of cell wall saccharification represents the major technical hurdle that must be overcome before the full vision of plant fuelled biorefinery can be realized.

The development of high throughput (HT) methods of screening for phenotypic and biochemical alterations in plants has played an important role in identifying the functions of genes and enzymes in specific pathways in plants and other organisms. However, the analysis of large populations of plants for cell wall digestibility is time consuming, labour intensive and expensive [8]. The availability of automated, sensitive and reliable methods to reveal differences in the digestibility of plant materials is essential for identifying and selecting genetic loci with the potential to improve the quality of lignocellulosic raw material [9].

Adaptation of biomass saccharification analysis to an HT format requires the miniaturization of processes. In most of the configurations for the conversion of lignocellulosic biomass into sugars and other bioproducts, the biomass is first pretreated, then hydrolyzed, and finally the resulting sugars are used for fermentation or other chemical transformations [10]. Therefore, a reliable analysis of saccharification properties should be able to reproduce these steps on a smaller scale to evaluate the saccharification in large and diverse sample populations. One of the major challenges in developing such a system derives from the fact that lignocellulosic biomass is an insoluble and heterogeneous material that is difficult to handle at the milligram scale.

Handling and distribution of the biomass materials is facilitated by grinding into very small particles. However, size reduction represents the first step in the conversion process, and is in itself a pretreatment that may increase digestibility. Decker *et al*. [9] reported that milling material down to 20-80 μm mesh particle size does not affect digestibility. However, reducing particle sizes below that threshold increases saccharification, and might therefore mask potential differences in digestibility between genetically different materials. At present, a number of alternatives have been used for dispensing biomass in an accurate and reproducible manner, ranging from the distribution of fabricated sheets of lignocellulose in microplate wells, to pipetting biomass slurry [11,12].

Considerable resources have been invested in HT screening systems to identify the optimum pretreatments, and there is much information available about this crucial aspect of biomass conversion [9,13]. This information is essential to assist the development of efficient industrial platforms.

Improvement of feedstocks for conversion into biofuels is an equally challenging area, involving breeding crops with reduced recalcitrance to saccharification. What is required here is a simple and rapid assay that can be applied to biomass preparations to identify cell wall traits that facilitate the conversion of biomass. Indeed, examples of low throughput characterization of biomass for this purpose can be found in the literature [14,15], but such approaches lack the throughput required to evaluate large populations of plants.

In this paper, we present an analytical platform that can perform saccharification analysis in a 96-well plate format, and has been developed to allow the screening of lignocellulose digestibility of large populations of samples from varied plant species. We have scaled down the reaction volumes for gentle pretreatment, partial enzymatic hydrolysis and sugar determination, to allow large numbers to be assessed rapidly in an automated system. Our aim is to evaluate biomass samples from different plants to screen mutant and breeding populations and transgenic lines for variability in saccharification. Previous studies have shown that alterations in lignin content can have profound effects on biomass conversion [14,16]. Based on this knowledge, the HT analysis was validated using transgenic tobacco with altered lignin, and we demonstrate that it is a reliable and reproducible method for revealing differences in saccharification. This method can also be used to assess the relative efficacy of different enzyme mixtures used for biomass digestion.

## Results

### Overview of the platform and loading variability

#### Formatting of the plant materials in 96-well plates

**Figure 1:**
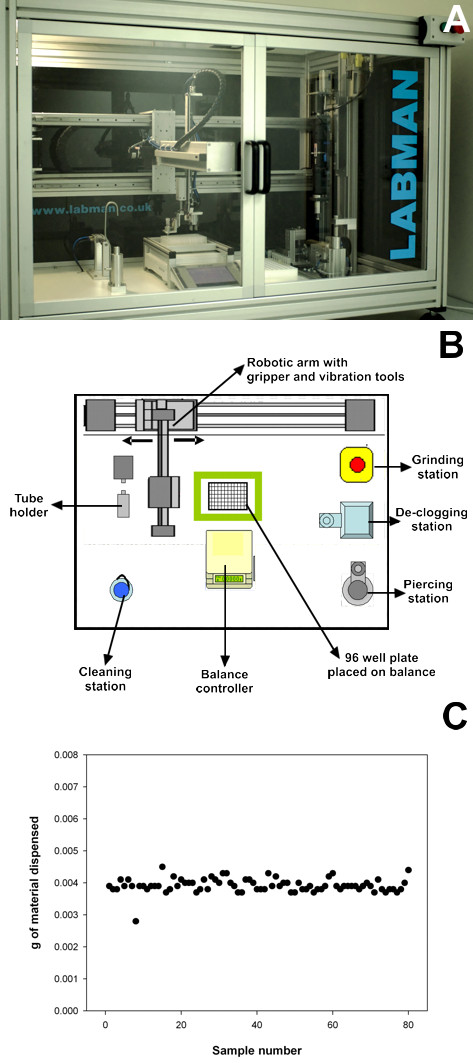
**Robotic platform for grinding, dispensing and weighing samples**. **(A) **General view of the robot; **(B) **schematic plan of the robot showing the different stations; **(C) **accuracy of the sample weights observed in the delivery of plant powder in a 96-well plate.

To obtain a precise and uniform distribution of the plant materials for the digestion assays, a robotic platform was designed to grind, distribute and weigh dry plant samples (Figure 1A). This device consists of a robotic arm that is able to move vials containing plant samples between six different stations (Figure 1B). The first step is a grinding station where the vials containing three ball bearings are shaken at approximately 5000 rpm. The desired particle size for each plant material can be achieved by varying the grinding time in this station. Once a suitable particle size is achieved, the grinding time is kept constant across all the samples of the experiment. The sample tubes are subsequently moved to a declogging station, where the powder is mixed by inverting the vials. At the third station, the base of the vial is pierced to allow dispensing of the powder into the designated locations in 96-well plates at the last station. Finally, the vials move to a 96-well plate placed on top of a balance, and the robotic arm vibrates at variable speeds to dispense the powder up to a determined sample weight, with the final weight recorded for each sample. The standard weight of each sample used in this paper is 4 mg, and the accuracy of dispensing is 0.1 mg (Figure 1C). The plates are then sealed with a silicone cover to avoid evaporation during the pretreatment and hydrolysis.

#### Pretreatment, hydrolysis and sugar detection

**Figure 2:**
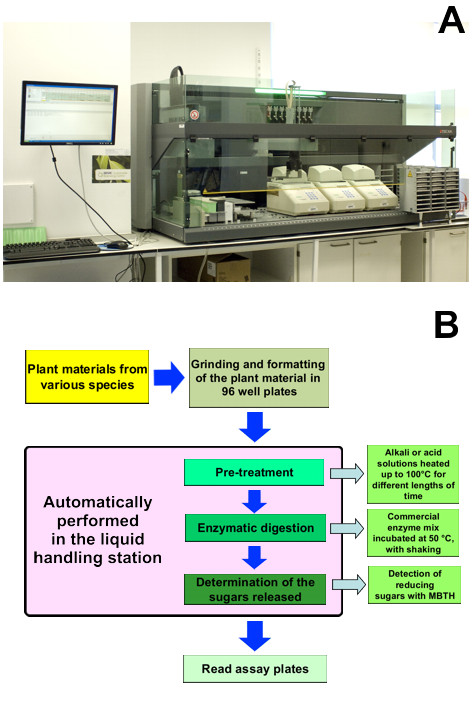
**Liquid handling station**. **(A) **General view of the liquid handling station; **(B) **flow chart showing the steps involved in the saccharification analysis.

**Figure 3:**
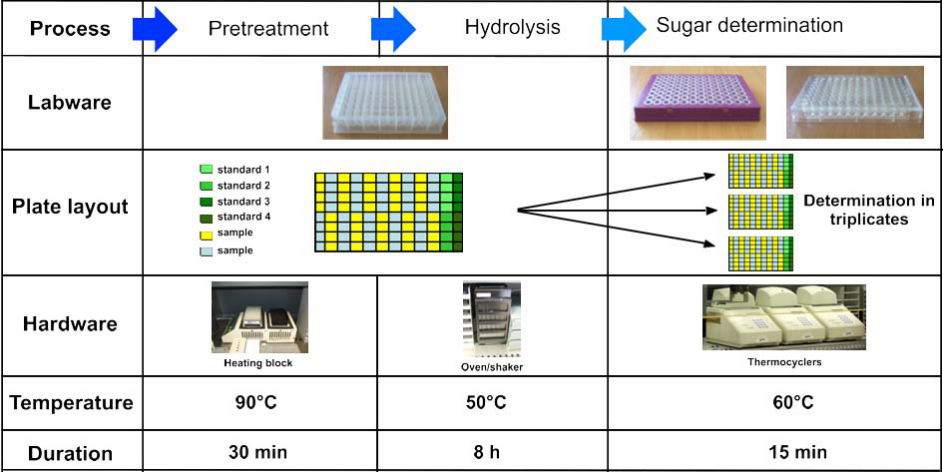
**Labware, hardware and incubation conditions used in the saccharification analysis**.

Once the samples are loaded as described above, the 96-well plates are processed by a robotic liquid handling system (Figure 2A). This system performs mild pretreatment of the plant materials by heating in the presence of acid or alkaline solutions, followed by removal of the pretreatment solution, enzymatic hydrolysis and quantification of the released material as reducing sugar equivalents (Figure 2B). It should be noted that the aim of this system is primarily to screen plant materials rather than to improve the pretreatment conditions on a pilot or industrial scale, and consequently only mild heating is applied to the biomass samples before hydrolysis as a form of pretreatment. The platform allows flexibility in the duration of all the step (Figure 3).

The pretreatment takes place on a heating block where the temperature can be regulated up to 100°C (Figure 3). The pretreatment is followed by several rinses (usually six) with a buffered solution, before the enzyme mix is dispensed into the wells. The rinses are carried out by adding buffer to the pretreatment solution, and subsequently removing 50% of the total volume after allowing the solids in the sample to settle. The height of the aspiration is set at half of the liquid mass to avoid aspirating solids from the bottom of the well; there is negligible loss of solids using this approach. These washes are repeated six times to bring the pH of the sample to the same value as that of the hydrolysis. The 96-well plates are then incubated at 50°C with constant shaking in ovens designed for delivering an even heat to such plates. After hydrolysis, aliquots of the digestate are removed from the plates for colorimetric assay of the released reducing sugars.

#### Detection of reducing sugars

A colorimetric method of sugar detection is used to reach the throughput required to analyze large populations. However, both the non-digested materials and the hydrolysate of selected samples can also be retained for further analysis as required. We assessed several methods for reducing sugar quantification, such as dinitrosalicylic acid (DNS) [17], bicinchonimic acid [18], 2-cyanoacetamide [19] and 3-methyl-2benzothiazolinonehydrazone (MBTH) [20]. MBTH was selected as the most suitable method, being the easiest to automate and the least susceptible to interference from compounds such as proteins. We modified the highly sensitive MBTH method of Anton and Barrett [20] for use on the robotic platform so that it could accurately quantify sugars at the concentrations present in the biomass hydrolysates, with a final volume of 250 μl, suitable for a standard optical 96 well plate. Table 1 shows a comparison of the reaction conditions between the original method and the modification for our HT assay.

**Table 1:** Summary of the changes from the original MBTH^1 ^method to be adapted to the HT^2 ^format

Reagent	Original method		Modification for HT assay	
	
	Volume of reagent^3^	Concentration^3^	Volume of reagent^4^	Concentration^3^
Sample	100 μl	-	75 μl	-

NaOH	100 μl	0.5 N	25 μl	1 N

MBTH mixture	100 μl	1.5 mg/ml MBTH 0.5 mg/ml DTT	50 μl	0.21 mg/ml MBTH 0.7 mg/ml DTT

Oxidising Reagent	200 μl	0.5% (FeNH_4_(SO_4_)_2_) 0.5% Sulfamic acid 0.25 N HCl	100 μl	0.5% (FeNH_4_(SO_4_)_2_) 0.5% Sulfamic acid 0.25 N HCl

H_2_O	500 μl	-	-	-

Heating	80°C for 15 min -		60°C for 20 min -	

Range of detection	0 to 20 nmol		0 to 200 nmol	

^1^HT = high throughput.

^2^MBTH = 3-methyl-2benzothiazolinonehydrazone.

^3^Anton and Barrett [20].

^4^HT determination.

**Figure 4:**
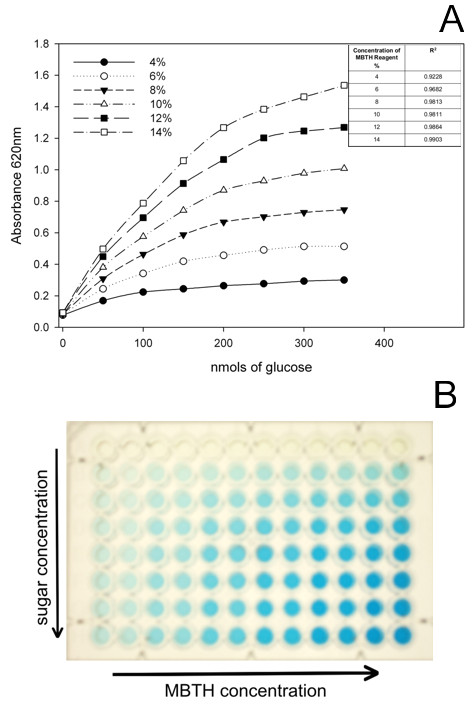
**Adaptation of the MBTH method for use in automated analyses (A)**. Calibration curve for the detection of reducing sugars using increasing concentrations of MBTH. Figure inset shows the R^2 ^values obtained for the different MBTH concentrations between 0 and 200 nmol.

The concentration of MBTH was optimized to increase the range of concentrations at which the detection of reducing sugars is linear. Figure 4 shows the linearity between different dilutions of MBTH reagent and a range of concentrations of glucose. In a similar manner, we optimized the temperature and incubation times to give the absorbance reading, resulting in incubation at 60°C for 15 min (data not shown).

**Figure 5:**
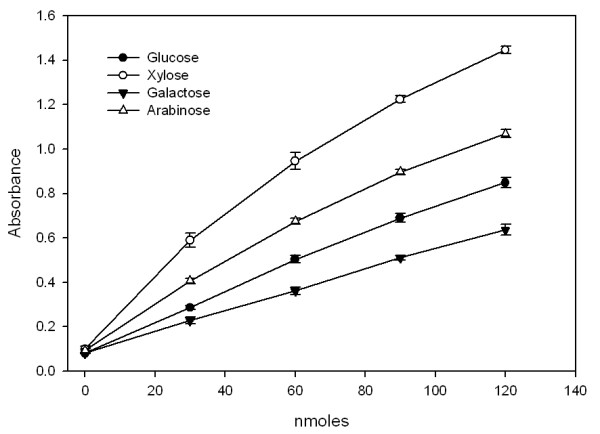
**Calibration curve of the MBTH detection of different monosaccharides**.

The sensitivity of the automated method with different sugars was established by measuring the response and linearity of determinations, which was carried out by comparing the original method with that modified for the automated system. Figure 5 shows the calibration for glucose, xylose, arabinose and galactose. Although the response in absorbance units is different for each monosaccharide, the quantification is linear in the range of concentrations at which the saccharification analysis is performed in the automated platform (between 0 and 150 nmol). The varied response in detection by MBTH for different monosaccharides contradicts the report from Anton and Barret [20] regarding the use of the method for a range of sugars. We decided to rely on our measurement of reducing sugar equivalents as glucose equivalents based on standard glucose curves, as this is the major monosaccharide in the cell wall.

### Reproducibility of the saccharification assay

**Figure 6:**
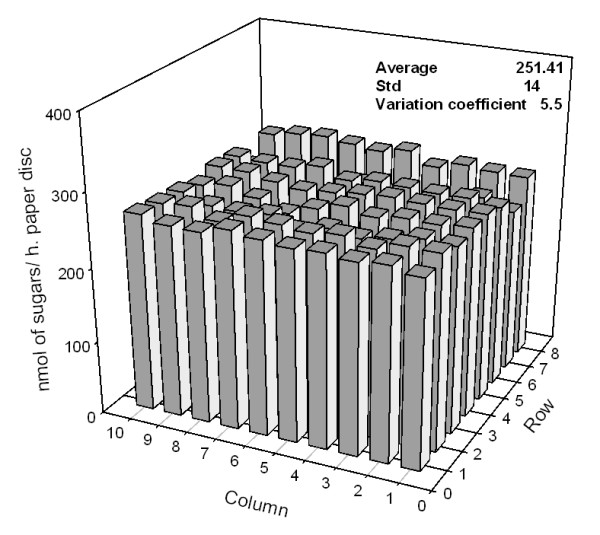
**Quantification of the released reducing sugars in the hydrolysis of paper discs in a 96-well plate**.

We used paper filter discs (Whatman No 1, Cole-Parmer Instrument Company, London, UK) to test the variability within the 96-well plate based automated assays. After pretreatment with 1% H_2_SO_4 _for 30 min at 90°C, the discs were digested in the robotic liquid platform for 8 hour, and the released reducing sugars determined in triplicate. The variation coefficient across 96 wells was 5.5% (Figure 6). This variation is considerably lower than the 9% variation observed when the saccharification of paper discs was performed manually (data not shown).

### Enzyme characterization using the robotic platform

**Figure 7:**
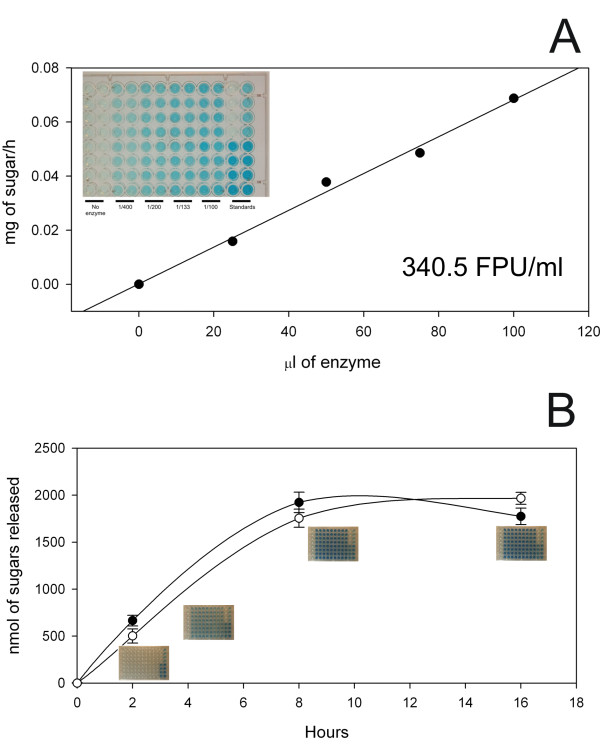
**Determination of cellulase activity using the automated assay**. **(A) **Sugar release from paper discs using increasing enzyme loadings. Image shows a typical plate obtained from the liquid handling station. **(B) **Time course of the release of reducing sugar equivalents from Whatman filter paper discs by two enzyme mixtures of equal activity.

The robotic station was designed with sufficient flexibility to allow the characterization of glysosyl hydrolases and of the digestibility of plant samples. We used this platform to determine the filter paper units (FPU) of enzyme mixtures, and to compare different enzyme mixtures against a diversity of substrates [21]. FPU were measured by the robot using equal amounts of filter paper (Whatman No. 1) different amounts of a typical mixture of commercial enzymes devised for biomass digestion (Celluclast and Novozyme 188, Novozymes A/S, Bagsvaerd, Denmark) (Figure 7A) The hydrolysis was carried out for 1 hour at 50°C, and the FPU calculated based in the amount of glucose released as detailed previously [19]. Under these conditions the logarithmic phase of the enzymatic reaction occurs at between 2 and 12 hours of hydrolysis. Figure 7B shows the time course of sugar release between equal enzyme loadings of two different enzyme mixtures

### Characterisation of altered lignin transgenic tobacco using the HT saccharification platform

To validate the analytical platform for detecting differences between different plant genotypes, we tested a set of transgenic tobacco plants in which expression of various lignin biosynthesis genes had been suppressed and the consequent changes in the content and structure of lignin had previously been characterized [22,23]. Line *ccr *had suppression of cinnamoyl-CoA reductase (CCR), line *cadccr *had suppression of both CCR and cinnamyl alcohol dehydrogenase (CAD), and line *cadcomt *had suppression of both CAD and caffeate O-methyltransferase (COMT). All three lines have reduced lignin, with 53%, 58% and 74% of wild type lignin levels, respectively.

**Figure 8:**
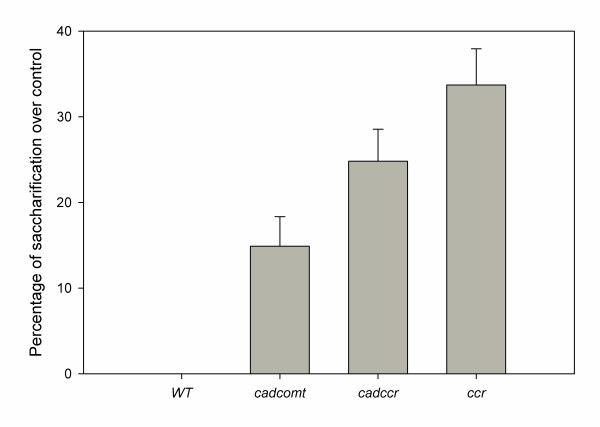
**Manual determination of the saccharification of lignin deficient tobacco**. Samples from wild type, a double mutant for cinnamyl alcohol dehydrogenase (CAD) and caffeate O-methyltransferase (COMT) (*cadcomt*), double cinnamoyl-CoA reductase (CCR) and COMT material (*ccrcomt*) and a CCR mutant line (*ccr*) were compared after 8 hours of hydrolysis. The samples were heated in the presence of 0.5N NaOH, and subsequently rinsed before the enzymatic digestion. The experiment was carried out in triplicate. Bars represent values ± SD.

We first tested the saccharification of powdered stems manually, using a similar protocol to the one performed by the robot with a 0.5N NaOH pretreatment at 90°C for 30 min. The modified lignin materials showed different degrees of saccharification compared with the wild type (Figure 8). The *ccr *and *cadccr *transgenics showed a 33% and 25% increase, respectively, in the amount of reducing sugar equivalents compared with wild type after 2 hours of incubation, whereas the *comt *mutant had a much smaller increase in sugar release. After 2 hours of hydrolysis, the wild type line released an equivalent of the 10% of the sugars contained in structural carbohydrates.

**Figure 9:**
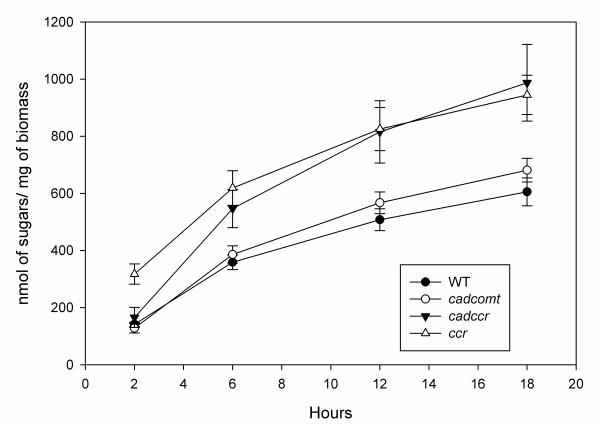
**Time course of the saccharification of lignin deficient tobacco material performed in the automated platform**. Samples from wild type, a double mutant for cinnamyl alcohol dehydrogenase (CAD) and caffeate O-methyltransferase (COMT) (*cadcomt*), double cinnamoyl-CoA reductase (CCR) and COMT material (*ccrcomt*) and a CCR mutant line (*ccr*) were loaded into 96-well plates and analysed using the high throughput platform. Sample aliquots from the hydrolytes were taken and assayed for reducing sugar equivalents after 2, 6, 12 and 18 hours. Results are representative of experiments performed in triplicate. Bars represent values ± SD of eight experiments.

We then used the automated platform to determine the release of reducing sugar equivalents from the same materials, over an 18 hour period (Figure 9). The results obtained using the automated platform showed similar differences in saccharification to those obtained by manual determination. Although after 2 hours of enzymatic hydrolysis only small differences between the wild type and the other three genotypes could be seen, the increased saccharification apparent in *ccr *and *cadccr *lines was significant after 6 hours and became more obvious as the digestion time increased (Figure 9). The sugars released by the wild type after 6 hours were equivalent to 15% of the total polysaccharide content in the tobacco stems. The *comt *lines also had an increased saccharification with respect to the wild type, but this difference only became significant after 18 hours of hydrolysis.

**Figure 10:**
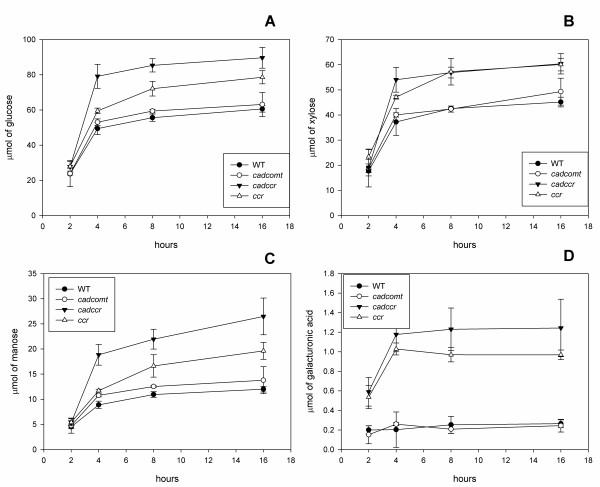
**Release of sugars from tobacco lignin mutants at different incubation times**. Samples from wild type, a double mutant for cinnamyl alcohol dehydrogenase (CAD) and caffeate O-methyltransferase (COMT) (*cadcomt*), double cinnamoyl-CoA reductase (CCR) and COMT material (*ccrcomt*) and a CCR mutant line (*ccr*) were loaded in 96-well plates and analysed using the high throughput platform. Samples were taken after 2, 4, 8 and 16 hours of incubation with hydrolytic enzymes. **(A) **Glucose, **(B) **mannose, **(C) **xylose and **(D) **galactose A were quantified by high performance anion exchange chromatography. Bars represent values ± SD of experiments carried out in triplicate.

In this automated system, the saccharification is evaluated by measuring the release of reducing sugars in the medium. We verified this method of analysis by running, in parallel, a determination of monosaccharides released during the hydrolysis in the same system. Figure 10 shows the evolution of glucose, xylose, galactose A and mannose, as these were the most abundant sugars released during an 18 hour digestion of the tobacco lignin transgenics. In general, the monosaccharide analysis confirmed the results obtained by measuring the release of reducing sugars using the modified MBTH method. The *ccr *and *cadccr *transgenics released a significantly higher amount of all four sugars Differences became more evident with the progression of the enzymatic digestion. Interestingly, the amounts of galactose A released from the *ccr *and *cadccr *mutants was approximately six times higher than that from the wild type. The results validate the saccharification analyses carried out in the HT platform compared with both the manual evaluation of digestibility and the use of reducing sugar equivalents to determine the extent of digestion, which is in broad agreement with more detailed analysis by high-performance anion-exchange chromatography of the released sugars.

## Discussion

We report a simple and robust method for HT screening of large populations of plants to monitor differences in saccharification as a marker for improving energy crops. This method focuses on the detection of different behaviors in sugar release during enzymatic hydrolysis. We have developed a platform that formats the biomass material in 96-well plates with minimum error on a milligram scale. This is an essential requirement to bring the scale of the saccharification analysis down to a volume that allows an automated, HT process.

We decided to limit the range of temperatures at which the pretreatments are conducted, as the aim of the work was to develop an assay capable of detecting differences in lignocellulose digestibility between plant varieties, in order to identify genes that may be used to improve plant feedstocks. The robotic platform can deliver different pretreatments at less than 100°C and at ambient pressure. Thus, our approach differs substantially from that described by Studer *et al*. [13] for evaluating industrially relevant pretreatment conditions. If necessary, however, it is possible to use harsher pretreatment conditions on the biomass samples before loading them in the automated platform, similar to approaches described for other HT systems for biomass analysis [12,13]

One of the major features of the system is its flexibility, allowing several other uses for the platform besides the standard saccharification analysis. For example, we have used the system to determine the kinetics of enzyme preparations. We have determined the activity of cellulases and have compared a matrix of different enzymes and substrates (data not shown). Another interesting adaptation of the system is its use in evaluating plant materials expressing heterologous glycosyl hydrolases such as cellulases (data not shown). In this process, the plant materials are incubated at optimum conditions for the activity of the heterologously expressed enzymes, then a pretreatment is performed and subsequently the materials are saccharified with exogenous enzyme. Determination of the released reducing sugars is performed after each step of the procedure.

One of the main challenges faced in the miniaturization of the process was the delivery of uniform heating in the different types of 96-well plates used for the various steps. The volume and duration of the pretreatment (350 μl for 30 min) did not represent a challenge, as a heating block was sufficient to provide this (data not shown), and the enzymatic hydrolysis at 50°C was carried out in incubation ovens (Figure 2A). However, the development of conditions for the detection of reduced sugars proved more challenging, as very small differences in temperature between wells produced large differences in the development of colour when using MBTH, and neither ovens nor heating blocks produced satisfactory results. We initially tried optical 96-well plates that could then be read directly in the plate reader. Using these plates, we attempted to heat the colorimetric reactions of the MBTH reaction in an oven. However, in this configuration, the sides of the plates heat up more rapidly than the centre, resulting in uneven colour development. Similarly, when we tried heating optical plates on flat heating blocks, the opposite problem was experienced, with the centre of the plates heating up more rapidly than the sides. This issue was finally resolved by using thermocyclers with motorized lids to heat the reactions before adding the oxidizing reagent.

We designed the process to avoid the need for filter separation of solids from the supernatant of the enzymatic reactions. This was achieved by delivering the solids, using the grinding-dispensing robot, and subsequently pretreating in deep 96-well plates. The removal of the pretreatment solution is performed by successive additions and removals of buffer. The liquids are removed by positioning the pipettes above the level of the solids and leaving a dead volume of liquid after aspirating, o avoid disturbing the insoluble material. The successive aspiration of liquid is carried out after a time lapse to allow the solids to settle at the bottom of the wells.

Reducing sugar assays are performed in triplicate to ensure reduction of variability due to technical errors in the determination. Similarly, in every plate we include solution blanks and glucose standards used in the calibration curve as an internal control for the reaction.

In addition to the results have shown here for tobacco, we also analysed materials from *Arabidopsis, Brachypodium*, barley, maize, poplar and sorghum. Statistical analysis of the results showed that the number of replicates needed differs for each type of material, depending on the homogeneity in particle size that can be reached during grinding. In a standard plate, we typically use 16 wells for blanks and standards, leaving 80 wells for samples. For analysis of large populations, we run samples in quadruplicate, meaning that 20 samples can be included in one 96-well plate.

The MBTH based HT assay for the determination of the educing released from biomass materials after enzymatic digestion was chosen for adaptation to automation. DNS is widely used in manual determinations; however, its requirement of high temperatures (100°C) for colour development and the presence of phenol in the DNS reagent make this method difficult to adapt to standard liquid handling equipment. Similarly, the BCA and Nelson-Somogyi methods have disadvantages, as they produce a high background due to reactivity of the detection reagents with the enzymes added to the hydrolysis.

We adapted the MBTH method for rapid detection of sugars by changing the amount of reagent added and reducing the incubation temperature from 80°C to 60°C. The colour development in this assay is stable, and the plates can be read up to 3 days after being prepared. By comparing the original method with the modified automated system, we were able to show that even though the sensitivity of the manual method for detecting different sugars is higher, the automated system has a larger range of concentrations at which quantification is linear. The released sugars during hydrolysis can also be analyzed for simple sugars or oligosaccharides using standard high performance liquid chromatography or mass spectrometric methods as previously described [24].

We have shown that our automated system reliably detects differences in the saccharification of woody plant tissues from tobacco plants with previously characterized changes in lignin content. Indeed, the levels of saccharification after 2 hours of incubation correlated positively with the lignin content of the different lines, with *ccr*, the line with lowest levels of lignin, showing the highest saccharification, and *cadcomt*, with little reduction in lignin content, showing only moderate increases in saccharification. These data illustrate the significant effect that lignin levels play in determining the ease with which sugars can be released from cell wall polysaccharides, as reported previously [14,25].

## Conclusion

The HT saccharification analysis presented here was developed to assist in the evaluation of biomass material for release of fermentable sugars. The system is based on a 96-well format, and the digestibility is evaluated by the quantification of reducing sugars using a MBTH based technique. We adopted the use of an automated dispenser of biomass powder to overcome the difficulties of handling insoluble and heterogeneous plant materials. The core of the analysis is performed automatically, including mild pretreatment of the materials, hydrolysis and sugar determination. The use of tobacco lines deficient in lignin to test the system underlines both the reliability and sensitivity of the system to detect differences in saccharification, and the importance of lignins in the digestibility of lignocellulose.

These results provide a firm basis for subsequent use of this platform to characterize and compare the digestibility of mutant and transgenic plants and to broaden our understanding of the features that determine this parameter. The platform will also serve as a screening tool to identify plants from mutant and breeding populations to identify genes and molecular markers that can be used to improve digestibility in the woody biomass of crops such as maize, or dedicated biomass crops such as switch grass or miscanthus. We are currently using this system for both forward and reverse genetics based analysis of populations of *Arabidopsis*, *Brachypodium*, poplar, maize and barley.

## Methods

### Plant materials

Transgenic tobacco plants (*Nicotiana tabacum *cv Samsun) were produced by *Agrobacterium tumefaciens *mediated transformation The *ccr *plants were produced by disc transformation as described in Halpin *et al*. [26] and the *cadccr *and *cadcomt *lines were produced by transformation of tobacco seedlings using the method of Tinland *et al *[27].

Plants were grown in 10 inch pots containing general purpose compost (Universal Extra, Scotts Professional, Paper Mill Lane, Ipswich, UK). The glasshouse temperature was maintained at 30°C during a 16 hour day, and 25°C during the night. Lighting was provided by high pressure sodium vapor lamps (Powertone SON-T AGRO 400W; Philips Electronics UK Ltd, Guildford, UK), giving a light intensity of 250 μmol/cm^/^s. The humidity was in the range of 65 to 70%. Plants were grown for 10 weeks before the stem wood was harvested for analysis.

### Grinding and formatting of plant materials

Grinding and loading of plant powder into 96-well plates was performed using a custom-made robotic platform (Labman Automation, Stokesley, North Yorkshire, UK). The robot picks up individual 2 ml vials pre-loaded with plant material and ball bearings, and places them in the grinding station. The sample is then ground for a defined amount of time, and the vial is then declogged and pierced. Powder is then dispensed into the designated output wells in the 96-well plate. The standard target weight of material dispensed in these experiments was 4 mg. The final weight of material placed in each well is determined to the nearest 0.10 mg and automatically recorded. The powder feeding station is then moved to a vacuum cleaning station, where the nozzle is cleaned before the next vial is processed. This process is repeated until all 96 positions in the plate are complete. It is possible to configure the process for different numbers of replicates and samples. The robot can also be used for grinding only or feeding only.

### Automated liquid handling station

Pretreatment, hydrolysis and sugar determination are performed automatically by a robotic platform (Tecan Evo 200; Tecan Group Ltd. Männedorf, Switzerland), equipped with a liquid handling arm and a robotic arm for the movement of plates. A heating block (Torrey Pines Scientific, Carlsbad, USA) is integrated into the workbench to deliver pretreatments. The hydrolysis is performed in a monitored shaking incubator (Tecan Group Ltd.) which has a capacity for four plates. Heating for determination of reducing sugars is delivered in three thermocyclers with motorized lids (Bio-Rad Laboratories Ltd., Hemel Hempstead, UK)

### Hydrolysis of materials

Enzymatic hydrolysis was carried out using an enzyme cocktail with a 4:1 ratio of Celluclast and Novozyme 188 (cellobiase from *Aspergillus niger*) (both Novozymes, Bagsvaerd, Denmark). The enzymes were filtered using a Hi-Trap desalting column (GE Healthcare, Little Chalfont, Buckinghamshire, UK) before use. Hydrolysis was carried out during the indicated times, at 30°C in 25 mM sodium acetate buffer at pH 4.5.

### Sugar determination

#### Manual

Determination of sugars released after hydrolysis was performed using a modification of the method by Anton and Barrett [20] using 3-methyl-2-benzothiazolinone hydrozone (MTBH). Determination was carried out in a final volume of 1 ml using 300 μl of sample. The detection reaction contained 0.25 N NaOH, 3 mg/ml MTBH and 1 mg/ml dithiothreitol (DTT), and colour was developed after incubation at 70°C for 20 minutes by adding the oxidising reagent (0.2% FeNH_4_(SO_4_)_2_, 0.2% sulfamic acid and 0.1% HCl). Standard reactions of 50 nmol, 100 nmol and 150 nmol glucose were included, and the absorbance of the reaction was determined at 620 nm. This method was tested for detection of a range of sugars that are released from the cell wall, and showed sensitive detection of several monosaccharides.

#### Automated

The automated method for determination of reducing sugars was performed as described above with further modifications. The final volume of the reaction was 250 μl, using 75 μl of hydrolyzate. The colorimetric reaction was carried out by adding 25 μl of 1N NaOH and 50 μl of a solution containing 0.43 mg/ml MBTH and 0.14 mg/ml DTT. This mixture was heated at 60°C for 20 min in a thermocycler, and subsequently 100 μl of oxidising reagent was added. Each plate contained standard reactions of 50 nmol, 100 nmol and 150 nmol glucose. Three independent determinations of the reducing sugars were carried out for each saccharification reaction. The optical plates were read at 620 nm.

### Time course of the saccharification in lignin deficient tobacco lines

The powdered tobacco samples were pretreated with 0.5N NaOH at 90°C for 30 min. After pretreatment, the biomass was rinsed six times with 500 μl of Na Acetate buffer, a volume shown to be sufficient to remove interfering chemicals, before the enzymatic hydrolysis. After this the samples were incubated with shaking at 50°C in the presence of enzyme cocktail described above. Enzyme loading in the hydrolysis of all tobacco samples was 6.3 FPU/g of material. At the times indicated, an aliquot was taken from the 96-well plates, and then the plates were returned to the incubator. The entire procedure was automatically performed by the liquid handling station.

### Monosaccharide analysis

Monosaccharide analyses were performed by high performance anion exchange chromatography (HPAEC) (Carbopac PA-10; Dionex, Camberley, Surrey, UK) as described previously [28]. Samples (1.4 ml) were collected immediately after pretreatment and filtered using an ion exchange column (Dowex 1 × 2, Bio-Rad Laboratories, 32^nd ^& Griffin Ave, Richmond, CA, USA) to eliminate the H_2_SO_4 _from the samples. The monosaccharide standards used for quantification were arabinose, fucose, galactose and galactose A, glucose, mannose, rhamnose and xylose.

## Competing interests

The authors declare that they have no competing interests.

## Authors' contributions

LDG carried out the monosaccharide analysis, supervised the work in the laboratory, and contributed to the design of the robots, design of the experiments and manuscript preparation. CW carried out the preparation of materials and the saccharification analysis, and participated in the set up of the automated platform and manuscript preparation. AB generated and characterized the transgenic tobacco lines. CH supplied the tobacco materials and revised the manuscript. SJMM helped in the experimental design, participated in the design of the platform, contributed to the manuscript preparation, and obtained funding for the work. All authors read and approved the final manuscript.
